# Clinical prognostic factors for pediatric extra-abdominal desmoid tumor: analyses of 66 patients at a single institution

**DOI:** 10.1186/s12957-018-1536-x

**Published:** 2018-12-18

**Authors:** Bo Ning, Na Jian, Ruixue Ma

**Affiliations:** Department of Pediatric Orthopaedic, Children’s Hospital of Fudan University, 399 Wanyuan Road, Shanghai, 201102 China

**Keywords:** Desmoid tumor, Pediatrics, Prognostic factors, Recurrence, Surgery

## Abstract

**Background and purpose:**

Pediatric desmoid tumor (PDT) is rare and has a high local recurrence rate. The purpose of the present study was to analyze clinical risk factors of local recurrence in PDT patients.

**Materials and methods:**

We reviewed clinical data of 66 PDT patients from 2004 to 2015. All patients underwent macroscopically complete resection, and some recurrent tumors were prescribed radiotherapy. Factors such as sex, age at presentation, location, and proximity to nerves or vasculature were analyzed. The local recurrence rate and recurrence-free survival were analyzed with these factors.

**Results:**

All patients in the present study were children and had extra-abdominal tumors. The median follow-up time was 6.6 years. Thirty-six (55%) patients had local recurrence. Age, sex, tumor site, tumor size, and proximity to nerves/vasculature had a significant impact on prognosis in univariate analysis. Radiotherapy decreased the local recurrence rate. In multivariate analysis, younger age, tumor location in buttocks, larger tumor, and proximity to important nerves/vasculature were independent risk factors for poor prognosis.

**Conclusions:**

Favorable therapeutic strategies could be selected according to the preoperative prognostic risk factors. Radiotherapy should be considered for local recurrence of PDT.

## Background

Desmoid tumor (DT), as previously named aggressive fibromatosis, is a rare soft tumor with a high rate of local recurrence and inability to metastasize [1, 2]. DT is a fibroblastic neoplasm that is derived from musculoaponeurotic structures. The annual incidence of this tumor is 2–4 per 1,000,000 persons with a peak incidence in women after pregnancy and abdominal location [3]. In our pediatric institute, we found that pediatric DT (PDT) was most commonly located in the buttocks and lower extremities [4].

According to previous studies, PDT has a higher local recurrence rate and is more difficult to control when compared with adult DT (ADT) [5, 6]. Patients with PDT have a higher incidence of local recurrence after surgery and different clinicopathological changes compared with adult patients [7, 8]. Some studies have shown that more gene mutations and higher gene mutation rate are observed in PDT compared with ADT [8]. So, the different genes and clinical phenotypes indicate that PDT may be a different disease from ADT.

Although DT is a benign tumor, its treatment is still a challenge because of the high local recurrence rate, especially in children [2, 7, 9]. Many treatments can be selected including surgery, radiotherapy, chemotherapy, and wait-and-see. However, each therapeutic option has its weaknesses and complications, especially in children [2, 7]. Data for adult patients are sufficient, and there are some guidelines for diagnosis and treatment and follow-up recommendations [10–12]. However, it has been shown that PDT is more aggressive and difficult to treat than ADT [7, 13]. The data about treatment of PDT is limited, and prognostic factors are unclear.

In the present study, all patients were children, with extra-abdominal and deep tumors. The purpose of our study was to determine the prognostic factors for recurrence-free survival (RFS) in PDT at a single institution after macroscopic complete surgical resection.

## Methods

### Patients

We retrospectively reviewed 66 patients who underwent complete resection of histologically confirmed DT at Children’s Hospital of Fudan (CHFU) from 2004 to 2015. Superficial, intra-abdominal, and abdominal DTs were excluded. All patients were children with extra-abdominal and deep DTs. The remaining 66 patients were included in the study (Table 1). This study was approved by the Institutional Review Board at Fudan University and the guardian of each patient.

**Table 1:** Characteristic of 66 patients according to tumor presentations

Characteristic	Overall (*n* = 66)	Primary	Recurrent	*P* value
Total	66	36 (54.5%)	30 (45.5%)	
Gender				0.131
Male	42 (63.6%)	16 (38.1%)	26 (61.9%)	
Female	24 (36.4%)	14 (58.3%)	10 (41.7%)	
Age(years)				0.02
0–5	16 (24.2%)	5 (31.3%)	11 (68.7%)	
6–10	38 (57.6%)	16 (42.1%)	22 (37.9%)	
11–15	12 (18.2%)	8 (66.7%)	4 (33.3%)	
Location				0.001
Extremity	12 (18.3%)	5 (42.7%)	7 (58.3%)	
Trunk	16 (24.2%)	14 (87.5%)	2 (12.5%)	
Buttock	38 (57.5%)	10 (26.3%)	27 (73.7%)	
Size (cm)				0.003
0–5	21 (31.8%)	16 (76.2%)	5 (23.8%)	
5–10	31 (47.0%)	9 (29.8%)	22 (70.2%)	
> 10	14 (21.2%)	5 (35.7%)	9 (64.3%)	
Adjacent to nerves/vascular				0.001
Yes	25 (37.9%)	5 (20.0%)	20 (80.0%)	
No	41 (62.1%)	25 (60.9%)	16 (39.1%)	
Radiotherapy				0.012
Yes	27	20 (74.1%)	7 (25.9%)	
No	66	30 (45.5%)	36 (54.5%)	

### Data collection

The following clinical characteristics were prospectively recorded: sex, age at diagnosis, tumor site, tumor size, proximity to important blood vessels or nerves, and last follow-up date. All tumors were deep and extra-abdominal. Tumor site was categorized as buttocks, trunk, or upper or lower extremities. Tumor size was determined by radiological measurement and pathological report and divided into three groups (0–5, 5–10, and > 10 cm) according to previous studies [7, 9, 14]. The relation between tumor and important blood vessels or nerves was observed during surgery (Table 1). Histological slides of all patients were rechecked by two pathologists at CHFU, and all tumor margins were clear. Local recurrence was the endpoint and was defined by clinical examination and magnetic resonance imaging.

### Statistical analysis

Data were analyzed by SPSS for Windows, version 16.0 (Chicago, IL, USA). Chi-square or Fisher’s exact test was used for comparison of categorical variables. The Kaplan-Meier method was used to analyze RFS of patients, and comparisons were analyzed by the log-rank test. Univariate and multivariate analyses were performed by Cox regression analysis. Two-sided *P* < 0.05 was considered statistically significant.

## Results

### Patient characteristics

The characteristics of the 66 patients are detailed in Table 1. There were 42 boys (63.6%) and 24 girls (36.4%), with a median age of 7.5 years (range 0.3–14 years). The average duration of follow-up was 6.6 years (range 2–11.8 years). The median tumor size was 11.2 cm (range 4–30 cm). Tumors were located at the following sites: buttocks (38, 57.5%), extremities (12, 18.3%), and trunk (16, 23.2%). A total of 36 patients had local recurrence.

### Patient management

**Fig. 1:**
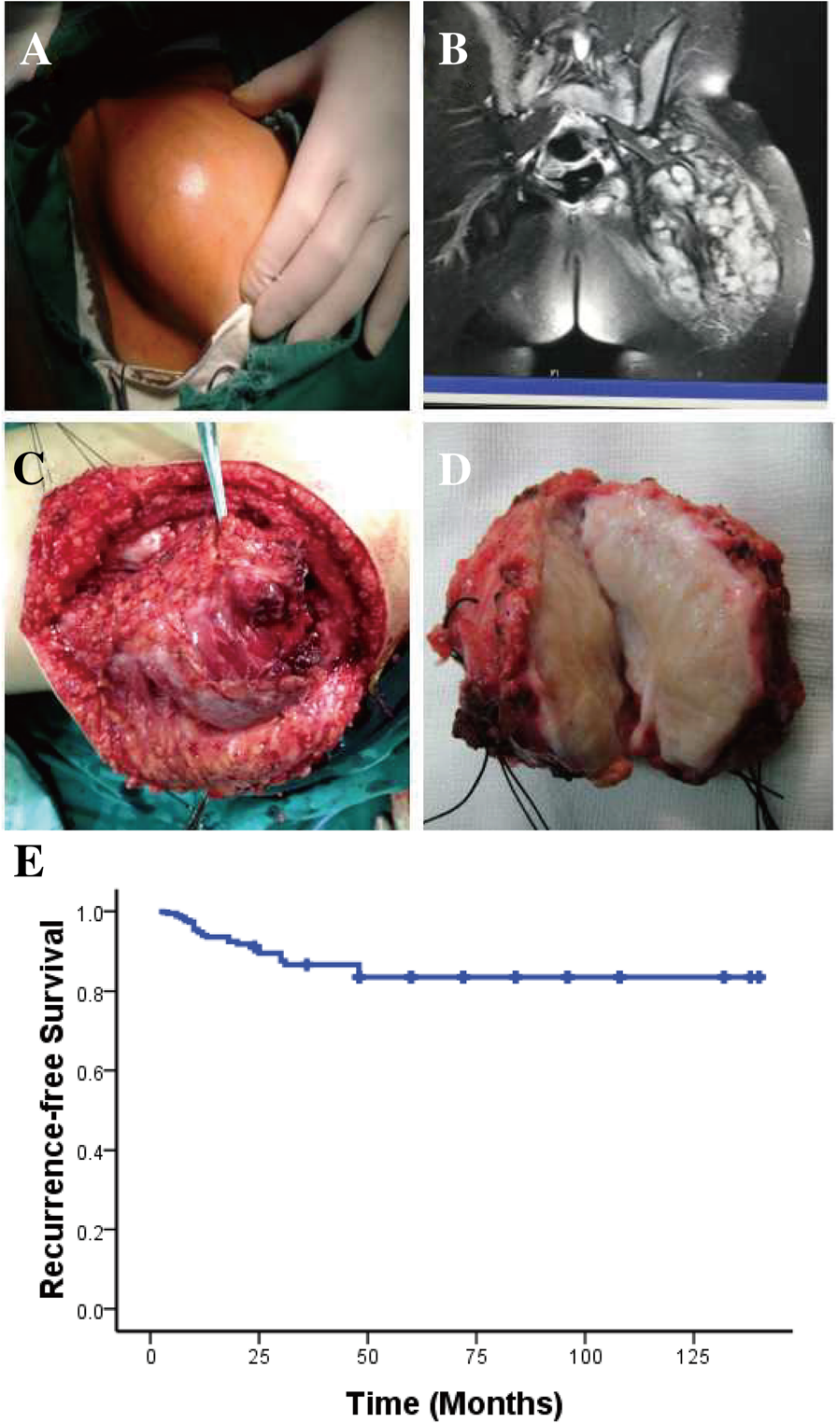
**a** Preoperative appearance of the tumor. **b** Magnetic resonance imaging of the left buttock. **c** Tumor during operation. **d** The tumor underwent macroscopic complete resection. **e** RFS in 66 patients analyzed by the Kaplan-Meier method

All patients with tumor symptom such as pain, huge bump, or joint limitation underwent complete surgical resection of PDT with clear margins after operation. The primary tumor underwent surgical resection alone (Fig. 1a–d), and 27 recurrent tumors underwent repeat surgery and radiotherapy. No patients were treated with chemotherapy. All patients underwent conservative resection, and none underwent amputation. Thirty-six patients had local recurrence and required at least one additional operation. We found that recurrent tumor was more closely related to the bone and nerves and resection was difficult. This indicated that the tumor became more aggressive after local recurrence. Nine patients experienced postoperative mild claudication that recovered after 3 months. Only one patient suffered from sciatic nerve palsy and recovered within 6 months after physical therapy. Given the complications of radiotherapy in children, no primary tumors received radiotherapy. For the 36 recurrent tumors, 27 patients underwent reoperation and postoperative radiotherapy. Doses ranged from 44 to 52 Gy according to the patient’s age and radiologist’s experience. The radiotherapic region is over 2 cm of the tumor. No complications of radiotherapy were observed during the time of follow-up.

### Patient outcome and prognostic factors

**Fig. 2:**
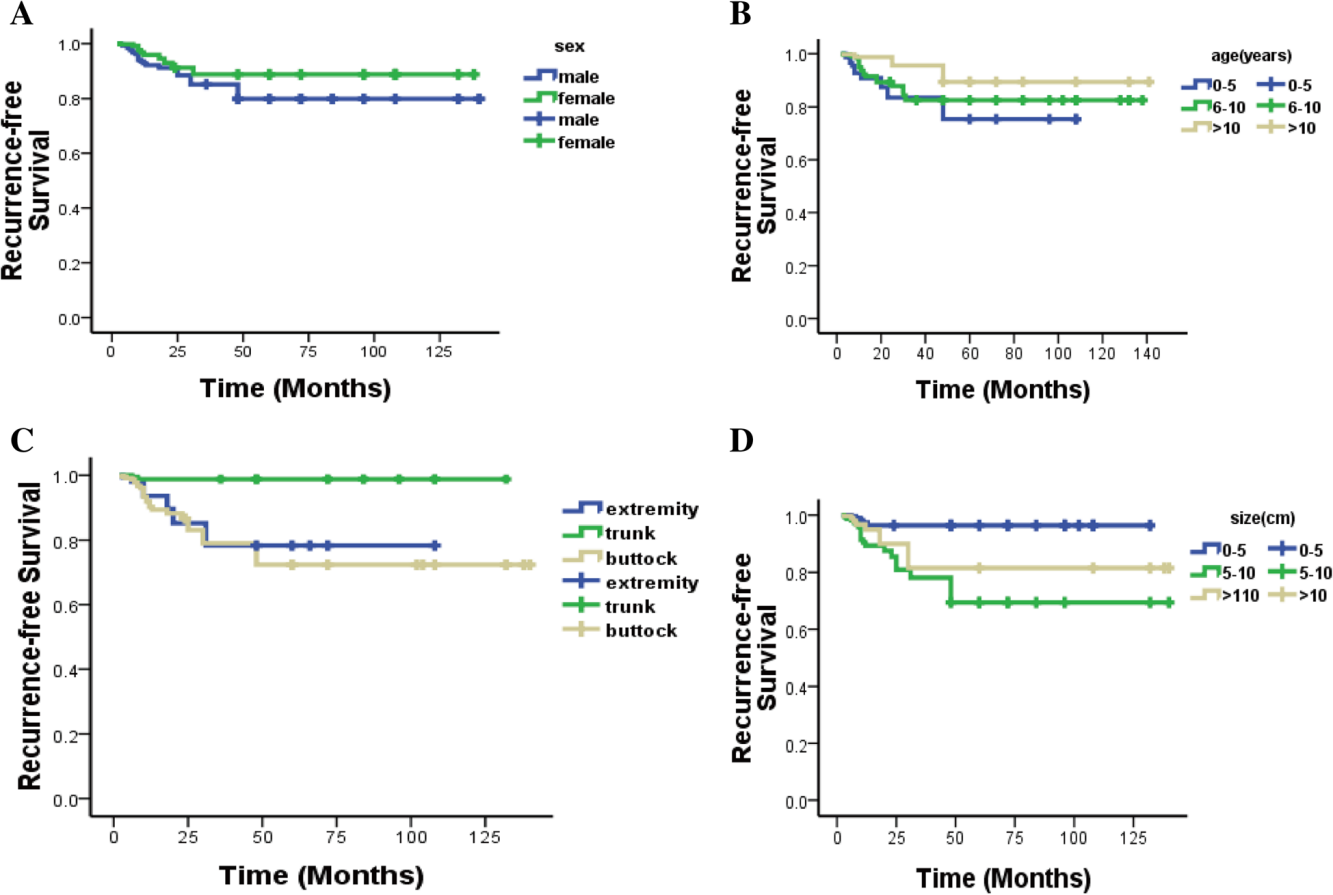
**a** RFS according to gender. **b** RFS according to age at tumor presentation. **c** RFS according to tumor location. **d** RFS according to tumor size

Thirty-six patients experienced local tumor recurrence at the time of last follow-up. The RFS rate was 45% (Fig. 1e). Several factors were analyzed for an association with local recurrence. Twenty-six of the male patients (61.9%) and 10 female patients (41.7%) had tumor recurrence (Table 1). There was no significant correlation between sex and recurrence (*P* = 0.131), but Kaplan-Meier analysis showed a high probability (*P* < 0.001) of recurrence in boys (Fig. 2a). The patients were divided into three age groups, 0–5, 6–10, and > 10 years. There was a peak local recurrence at ages 6–10 years (57.6%). There was no significant difference between the three groups (*P* = 0.162). However, two-by-two comparison indicated that older age was associated with lower recurrence rate (*P* = 0.02) (Table 1), and RFS also increased (*P* < 0.001) with older age (Fig. 2b). We separated the patients into three groups according to tumor location: buttocks, extremities, and trunk. Tumor recurrence was most frequent in the buttocks, followed by extremities, and the lowest recurrence rate was in the trunk (*P* < 0.001). RFS analysis showed that the highest local recurrence likelihood was in the buttocks (*P* < 0.001) (Fig. 2c).

**Fig. 3:**
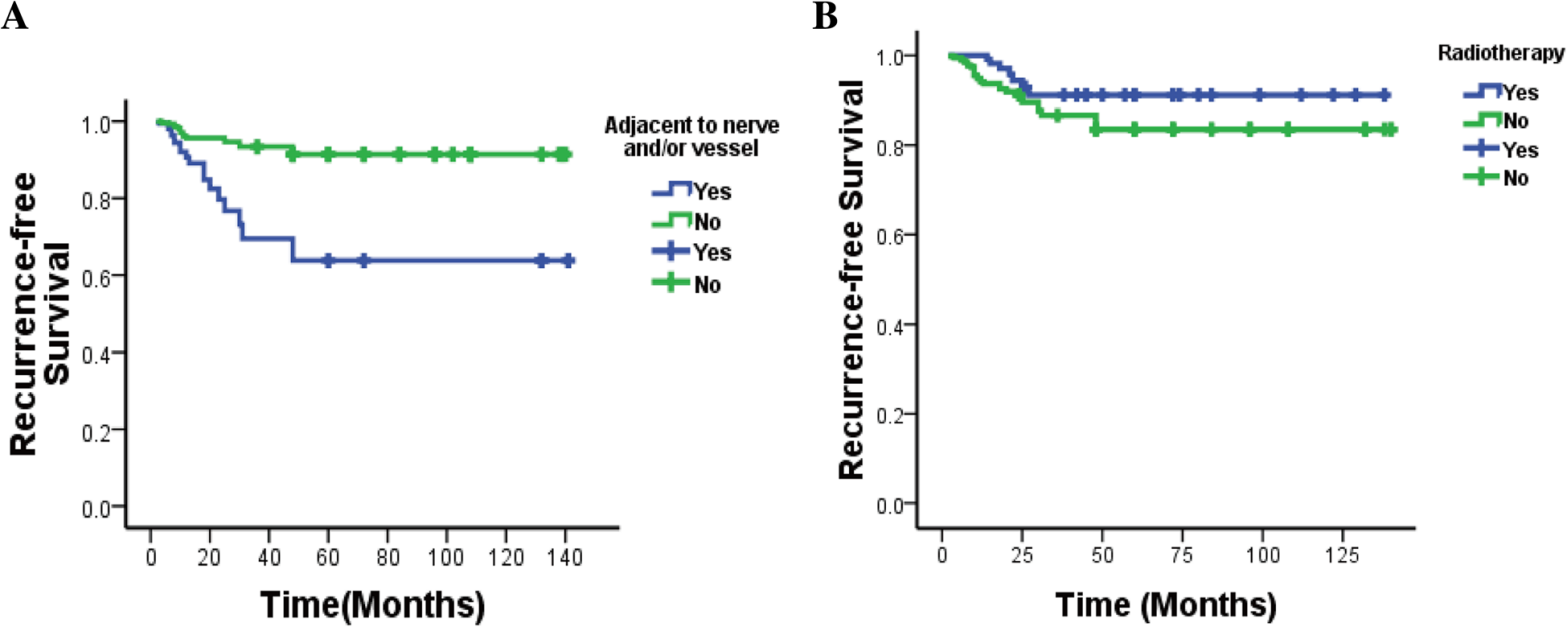
**a** RFS according to proximity of tumor to nerves and vasculature. **b** RFS with or without radiotherapy

We divided the patients into three groups according to tumor size: 0–5, 5–10, and > 10 cm. Analysis of tumor recurrence in each group showed that the greater the tumor, the greater the likelihood of recurrence (*P* = 0.003) (Table 1). RFS analysis also showed that the likelihood of tumor recurrence was less for small tumors (*P* < 0.001) (Fig. 2d). We divided patients into two groups according to tumor adherence to nerves and blood vessels: one group had close adhesion to nerves and blood vessels, and the other had no adhesion to these structures that were easily removed during surgery. There was a higher recurrence rate (*P* = 0.001) in children with tumors close to blood vessels and nerves (Fig. 3a).

All 27 patients who underwent radiotherapy had previous surgery and underwent reoperation after tumor recurrence, followed by postoperative radiotherapy to prevent further recurrence. After radiotherapy, there was still a recurrence rate of 25.9%, which was lower than that in the group who did not have radiotherapy (Table 1). Nevertheless, radiotherapy showed a decrease recurrence rate (*P* = 0.012) and survival analysis found that radiotherapy had the potential to delay recurrence (Fig. 3b). We did not observe any complications of radiotherapy during follow-up.

RFS was used to evaluate the effect of prognostic factors by univariate and multivariate analysis. Sex, age, location, proximity to important nerves/vasculature, and tumor size were independent prognostic factors (Table 2).

**Table 2:** Univariate and multivariate analysis of the effect of covariates of RFS for 66 patients

Factors	Univariate		Multivariate	
	HR (95% CI)	*P* value	HR (95% CI)	*P* value
Gender (male vs. female)	1.83 (1.15–2.22)	0.000	3.24 (2.56–4.09)	0.000
Age (vs. 0–5 years)				
6–10 years	2.70 (2.06–3.54)	0.000	10.53 (7.00–15.84)	0.000
> 10 years	1.78 (1.39–2.27)	0.000	3.24 (2.43–4.31)	0.000
Size (vs.0–8 cm)				
9–14 cm	0.19 (0.13–0.26)	0.000	0.22 (0.14–0.32)	0.000
> 14 cm	1.71 (1.40–2.10)	0.000	1.29 (1.01–1.65)	0.043
Adjacent to nerves/vascular (yes vs. no)	4.60 (3.85–5.48)	0.000	3.03 (2.34–3.84)	0.000
Location (vs. buttock)				
Extremity	27.80 (16.32–47.37)	0.000	5.78 (3.23–10.42)	0.000
Trunk	21.71 (12.40–38.01)	0.000	8.54 (4.62–15.79)	0.000

## Discussion

DT is also known as aggressive fibromatosis and is divided into three types: extra-abdominal, abdominal, and intra-abdominal; or superficial and deep-type fibromatosis according to anatomical position [4, 5, 10]. Although the tumor does not develop distant metastasis, it has a high local recurrence rate after operation, and so it has been defined as a local malignant tumor. Some studies have shown obvious differences in pathogenesis and pathogenic site [13, 15]. A higher recurrence rate has been observed in pediatric patients [4, 16]. In the present study, all patients were children, and abdominal and intra-abdominal types were excluded because of different pathogenesis, and a high local recurrence rate (55%) was observed. However, clinical prognostic factors in PDT are still unclear and controversial. The present study was focused on exploring the clinical prognostic factors of PDT.

The first factor we investigated was sex. Most studies have shown that DT appears mostly in women, and this is no different in children [8, 17, 18]. However, in the present study, male patients were more frequent than female patients. These differences may be explained as follows. (1) All patients were children, with different pathogenesis from adult patients. Our previous research showed that the mutation rate of three exons of *CTTNNB1* was higher in PDT than in ADT [4]. (2) Previous studies often included pediatric and adult patients, as well as all types of tumors [2, 14]. ADT most often occurs at abdominal and intra-abdominal sites in women after childbirth, and the prognosis is good [19]. The patients in the present study did not have these types of risk factors. Our study explored the relationship between sex and local recurrence. There was no significant sex difference in recurrence according to the *χ*^2^ test. However, male sex was one of the risk factors for bad prognosis according to survival and multiple-factor analyses. This result differed from previous studies [9, 11, 12, 18, 20] and may have been because we had pediatric patients with extra-abdominal tumors. Sex is an important risk factor during treatment and should be considered in patients with extra-abdominal PDT.

The relation between age and prognosis of DT is controversial [7, 9, 12, 19]. In most studies, younger age was an important risk factor for local recurrence [19, 21–23]. However, some studies have shown no relationship between age and local recurrence [9, 19]. In the present study, the younger patients had a higher recurrence rate. Although most studies and the present study showed that younger age at tumor presentation resulted in higher local recurrence rate, one study focused on pediatric patients suggested that older age increased postoperative local recurrence [7]. Patients in most other studies consisted of adults and children, and younger patients showed higher local recurrence rate. The reason may be that lower age group has some pediatric patients. Some authors have suggested that the high recurrence rate in younger patients is because pediatric patients have different and a higher number of gene mutations than older patients have [4, 8, 14, 24]. Moreover, DT cell proliferation decreased with older age. It is suggested that patients aged < 30 years need positive therapy with wide resection, additional therapy, and close follow-up [22, 25]. For older patients, less aggressive treatments such as partial resection, radiotherapy, and other less invasive methods, even wait-and-see, could be considered [26]. There have been few studies focused on pediatric patients, so the data for the prognostic effect of age for PDT are not sufficient. Our study suggested that age was an independent risk factor for local recurrence of PDT.

Tumor location is always an important risk factor for DT prognosis. There were obviously different groups in the present study in contrast to previous studies. In the present study, abdominal and intra-abdominal tumors were excluded. Extra-abdominal location, especially in the extremities, has been considered to be an independent risk factor that was associated with local recurrence of DT in previous studies [2, 7, 19, 23, 27]. PDT in the present study was divided into three groups according to location, including the buttocks, trunk, and upper and lower extremities. Tumors in the buttocks had poorest prognoses, and those in the trunk had the best. Moreover, PDT in the buttocks was most common in the present study and differed from adult patients in whom the tumor is often abdominal. These different clinical characteristics and prognoses suggest that tumors in different locations may have different biology or host-tumor site interactions. Different risk factors and location between pediatric and adult patients suggest that their tumors have different biological characteristics and are of different types [8]. The anatomical characteristics of the buttocks might be risk factors for higher recurrence. Tumors in the buttocks are often difficult to find by patients, so they may be large and deeply seated, even invading the pelvic cavity through the greater sciatic foramen, and adhering to the sciatic nerve. All of these factors may lead to tumor recurrence. PDT is more aggressive when it is adjacent to blood vessels and nerves, especially in the buttocks and lower extremities. These results confirmed that tumor location was a risk factor for recurrence. Tumors that are adjacent to blood vessels or nerves have poor prognoses that macroscopic tumor was completely resected and tumor cells may exist because of protecting important vessels or nerves during operation [28].

Tumor size was an important risk factor for prognosis. In the present study, the tumors were often large, especially in the buttock and lower extremities. The tumors were divided into three groups, and the results suggested that the larger tumors had a higher recurrence rate. Therefore, tumor size was a risk factor for poor prognosis. Previous studies have shown controversial results about tumor size. Most studies have suggested that large tumors are more aggressive, difficult to completely resect, adhesive to important blood vessels or nerves, and have a higher local recurrence rate [9, 19, 22, 29, 30]. However, some studies have shown that tumor size is not an independent risk factor for prognoses [7, 9, 23]. Tumor margin has been considered an important risk factor for prognosis. For large tumors adjacent to important blood vessels or nerves, complete resection is a challenge. It is difficult to define the tumor margin during surgery [30], although intraoperative frozen section examination is a reliable way. However, DT has a wide margin and intraoperative frozen sections have their limitations. Some studies have shown that tumor resection margin is not a risk factor for prognosis [7]. In the present study, we did not analyze the relation between tumor resection margin and local recurrence because all patients underwent macroscopic complete surgical resection.

Radiotherapy has always been controversial for treatment of DT, especially in children [7, 15, 17, 31]. Some studies have suggested that radiotherapy is effective for DT [15, 32–34]. However, other studies have shown that radiotherapy is an uncertain factor for prognosis [2, 23, 27, 35]. For pediatric patients, potential complications of epiphyseal injury should be considered because of immature bones [7]. In the present study, because of care taken by the radiotherapist, only 27 patients received local radiotherapy. The radiotherapy dose range of 48–52 Gy was decided by a surgeon and radiotherapist. The results showed that radiotherapy significantly reduced the tumor local recurrence rate. Although we did not observe any complications of radiotherapy at the latest follow-up, we should be vigilant for epiphyseal injury in further follow-ups. PDT should be evaluated carefully for all risk factors before radiotherapy.

Treatment of PDT is a major challenge because of the high local recurrence rate and lack of clinical data focused on pediatric patients. Some studies have shown that PDT has a wider spectrum of gene mutations than ADT has [8, 14, 24]. Our previous study has confirmed that PDT has a higher number of mutations in exon 3 of *CTNNB1* mutation than ADT, especially at the S45F site [4]. It has been demonstrated that gene mutation at S45F is related to tumor local recurrence. Gene mutation increases the difficulty of PDT treatment. We did not include this risk factor in the present study because of insufficient data about gene mutations in some patients. This was a limitation to the study and is a subject for further research.

There were some other limitations to the present study. First, it was a retrospective study. Second, the number of patients and time of follow-up were not adequate. Third, the therapeutic method was surgery and surgery plus radiotherapy, and chemotherapy was not prescribed.

All patients in the present study were children and had extra-abdominal type PDT. We evaluated the clinical characteristics and analyzed the risk factors for prognosis. The study could provide helpful advice to doctors who are formulating a therapeutic strategy for PDT patients.

## Conclusions

There are some clinical risk factors for prognosis in pediatric extra-abdominal desmoid patients including age at presentation, gender, tumor location, and tumor size. Favorable therapeutic strategies could be selected according to the preoperative prognostic risk factors. Radiotherapy should be considered for the patients with local recurrence of PDT.
